# Supplementary material for: Environmentally dependent wood density influences forest structure and dynamics in a demographic vegetation model
Source: Quant Plant Biol. 2026 Feb 9;7:e5. doi: 10.1017/qpb.2026.10038 (PMC13078103; doi:10.1017/qpb.2026.10038)
Supplement: Voss et al. supplementary material [file S2632882826100381sup001.docx]

Section 1: Supplementary methods

- 1. Observational data from the Tree Ring Database

*Table S1: All datasets from which latewood density was used for the function building. * marks the sites explored LPJ-GUESS*

| **Site** | **Species** | **Location** | **Elevation** | **Reference** |
| --- | --- | --- | --- | --- |
| FRAN032* | PCAB | 48.57 N, 8.00 E | 1080 m | Schweingruber, F.H. (2002-05-29): NOAA/WDS Paleoclimatology - Schweingruber - Aubure (F), EU-Pr. - PCAB - ITRDB FRAN032. NOAA National Centers for Environmental Information. https://doi.org/10.25921/cxg9-0412. Accessed 2023-11-13. |
| FRAN033* | PCAB | 47.98 N, 7.08 E | 230 m | Schweingruber, F.H. (2002-05-28): NOAA/WDS Paleoclimatology - Schweingruber - Vitrimont (F), EU-Pr. - PCAB - ITRDB FRAN033. NOAA National Centers for Environmental Information. https://doi.org/10.25921/aner-ma33. Accessed [date] |
| GERM041 | PCAB | 47.8 N, 8.08 E | 1200 m | Schweingruber, F.H. (2002-05-28): NOAA/WDS Paleoclimatology - Schweingruber - Schluchsee (D), EU-Pr. - PCAB - ITRDB GERM041. NOAA National Centers for Environmental Information. https://doi.org/10.25921/j37c-ge15. Accessed 2023-11-16. |
| GERM042* | PCAB | 48.08 N, 7.68 E | 440 m | Schweingruber, F.H. (2002-05-28): NOAA/WDS Paleoclimatology - Schweingruber - Kaiserstuhl (D), EU-Pr. - PCAB - ITRDB GERM042. NOAA National Centers for Environmental Information. https://doi.org/10.25921/t9pe-4v02. Accessed 2023-11-16. |
| GERM044 | PCAB | 47.83 N, 7.70 E | 390 m | Schweingruber, F.H. (2002-05-28): NOAA/WDS Paleoclimatology - Schweingruber - Sulzbach-Tal (D), EU-Pr. - PCAB - ITRDB GERM044. NOAA National Centers for Environmental Information. https://doi.org/10.25921/xxzp-3288. Accessed 2023-11-16. |
| GERM046 | PCAB | 47.80 N, 7.75 E | 930 m | Schweingruber, F.H. (2002-05-29): NOAA/WDS Paleoclimatology - Schweingruber - Sirnitz SW (D), EU-Pr. - PCAB - ITRDB GERM046. NOAA National Centers for Environmental Information. https://doi.org/10.25921/d48p-3s04. Accessed 2023-11-16. |
| GERM048 | PCAB | 47.83 N, 7.98 E | 1320 m | Schweingruber, F.H. (2002-05-28): NOAA/WDS Paleoclimatology - Schweingruber - Silberberg (D), EU-Pr. - PCAB - ITRDB GERM048. NOAA National Centers for Environmental Information. https://doi.org/10.25921/v4xz-ws80. Accessed 2023-11-16. |
| GERM049 | PCAB | 47.95 N, 8.02 E | 1330 m | Schweingruber, F.H. (2002-05-29): NOAA/WDS Paleoclimatology - Schweingruber - Feldberg (D), EU-Pr. - PCAB - ITRDB GERM049. NOAA National Centers for Environmental Information. https://doi.org/10.25921/9k7m-7b26. Accessed 2023-11-16. |
| GERM050 | PCAB | 48.03 N, 8.35 E | 880 m | Schweingruber, F.H. (2002-05-29): NOAA/WDS Paleoclimatology - Schweingruber - Villingen-S. (D), EU-Pr. - PCAB - ITRDB GERM050. NOAA National Centers for Environmental Information. https://doi.org/10.25921/cgz6-bp44. Accessed 2023-11-16. |
| GERM052 | PCAB | 47.85 N, 8.03 E | 1250 m | Schweingruber, F.H. (2002-05-29): NOAA/WDS Paleoclimatology - Schweingruber - Seehalde (D), EU-Pr. - PCAB - ITRDB GERM052. NOAA National Centers for Environmental Information. https://doi.org/10.25921/8eza-yx85. Accessed 2023-11-16. |
| GERM053 | PCAB | 47.85 N, 7.78 E | 490 m | Schweingruber, F.H. (2002-05-29): NOAA/WDS Paleoclimatology - Schweingruber - Münstertal (D), EU-Pr. - PCAB - ITRDB GERM053. NOAA National Centers for Environmental Information. https://doi.org/10.25921/5h9f-hw42. Accessed 2023-11-16. |
| GERM055 | PCAB | 47.78 N, 7.75 E | 940 m | Schweingruber, F.H. (2002-05-28): NOAA/WDS Paleoclimatology - Schweingruber - Sirnitz NE (D), EU-Pr. - PCAB - ITRDB GERM055. NOAA National Centers for Environmental Information. https://doi.org/10.25921/ncyx-e145. Accessed 2023-11-16. |
| RUSS022 | PCOB | 66.87 N, 65.63 E | 230 m | Schweingruber, F.H. (2002-05-28): NOAA/WDS Paleoclimatology - Schweingruber - Polar Ural (rezent) - PCOB - ITRDB RUSS022. NOAA National Centers for Environmental Information. https://doi.org/10.25921/jwv5-ge33. Accessed 2023-11-16. |
| RUSS030 | PCOB | 67.97 N, 88.92 E | 160 m | Schweingruber, F.H. (2002-05-29): NOAA/WDS Paleoclimatology - Schweingruber - Kulyumbe River - PCOB - ITRDB RUSS030. NOAA National Centers for Environmental Information. https://doi.org/10.25921/946p-y556. Accessed 2023-11-16. |
| RUSS031 | PCOB | 69.58 N, 90.50 E | 60 m | Schweingruber, F.H. (2005-04-12): NOAA/WDS Paleoclimatology - Schweingruber - Ozera Lama - PCOB - ITRDB RUSS031. NOAA National Centers for Environmental Information. https://doi.org/10.25921/s0q6-ga19. Accessed 2023-11-16. |
| RUSS032 | PCOB | 66.13 N, 71.67 E | 80 m | Schweingruber, F.H. (2002-05-29): NOAA/WDS Paleoclimatology - Schweingruber - Nadim River B - PCOB - ITRDB RUSS032. NOAA National Centers for Environmental Information. https://doi.org/10.25921/5fkb-5g29. Accessed 2023-11-16. |
| RUSS033 | PCOB | 66.08 N, 77.68 E | 30 m | Schweingruber, F.H. (2002-05-28): NOAA/WDS Paleoclimatology - Schweingruber - Yevoyakha River - PCOB - ITRDB RUSS033. NOAA National Centers for Environmental Information. https://doi.org/10.25921/e8rr-9559. Accessed 2023-11-16. |
| RUSS034 | PCOB | 66.82 N, 69.28 E | 90 m | Schweingruber, F.H. (2002-05-28): NOAA/WDS Paleoclimatology - Schweingruber - Shchuchye River - PCOB - ITRDB RUSS034. NOAA National Centers for Environmental Information. https://doi.org/10.25921/9s1b-6890. Accessed 2023-11-16. |
| RUSS037 | PCOB | 65.50 N, 7265 E | 100 m | Schweingruber, F.H. (2002-05-28): NOAA/WDS Paleoclimatology - Schweingruber - Kheygiyakha River - PCOB - ITRDB RUSS037. NOAA National Centers for Environmental Information. https://doi.org/10.25921/vaz9-9693. Accessed 2023-11-16. |
| RUSS038 | PCOB | 70.27 N, 103.52 E | 130 m | Schweingruber, F.H. (2002-05-28): NOAA/WDS Paleoclimatology - Schweingruber - Kotuy River - PCOB - ITRDB RUSS038. NOAA National Centers for Environmental Information. https://doi.org/10.25921/p19n-rd94. Accessed 2023-11-16. |
| RUSS039 | PCAB | 64.92 N, 42.50 E | 230 m | Schweingruber, F.H. (2002-05-28): NOAA/WDS Paleoclimatology - Schweingruber - Pinega - PCAB - ITRDB RUSS039. NOAA National Centers for Environmental Information. https://doi.org/10.25921/hek7-pa26. Accessed 2023-11-16. |
| RUSS040 | PCOB | 65.60 N, 50.63 E | 70 m | Schweingruber, F.H. (2002-05-29): NOAA/WDS Paleoclimatology - Schweingruber - Nonburg - PCOB - ITRDB RUSS040. NOAA National Centers for Environmental Information. https://doi.org/10.25921/spns-as72. Accessed 2023-11-16. |
| RUSS042 | PCOB | 66.22 N, 56.33 E | 65 m | Schweingruber, F.H. (2005-04-13): NOAA/WDS Paleoclimatology - Schweingruber - Shchely Bozh - PCOB - ITRDB RUSS042. NOAA National Centers for Environmental Information. https://doi.org/10.25921/hxnd-an11. Accessed 2023-11-16. |
| RUSS043* | PCAB | 63.43 N, 43.55 E | 120 m | Schweingruber, F.H. (2002-05-29): NOAA/WDS Paleoclimatology - Schweingruber - Voroney - PCAB - ITRDB RUSS043. NOAA National Centers for Environmental Information. https://doi.org/10.25921/evfa-8z62. Accessed 2023-11-16. |
| RUSS047 | PCOB | 64.25 N, 53.57 E | 70 m | Schweingruber, F.H. (2002-05-28): NOAA/WDS Paleoclimatology - Schweingruber - Kedvaran - PCOB - ITRDB RUSS047. NOAA National Centers for Environmental Information. https://doi.org/10.25921/pbkz-sg87. Accessed 2023-11-16. |
| RUSS049 | PCAB | 62.17 N, 44.42 E | 60 m | Schweingruber, F.H. (2002-05-28): NOAA/WDS Paleoclimatology - Schweingruber - Verhnaja Toima - PCAB - ITRDB RUSS049. NOAA National Centers for Environmental Information. https://doi.org/10.25921/wwv4-f975. Accessed 2023-11-16. |
| RUSS056 | PCOB | 66.88 N, 51.95 E | 35 m | Schweingruber, F.H. (2005-04-13): NOAA/WDS Paleoclimatology - Schweingruber - Charijaga - PCOB - ITRDB RUSS056. NOAA National Centers for Environmental Information. https://doi.org/10.25921/tb18-mw11. Accessed 2023-11-16. |
| RUSS057 | PCOB | 62.47 N, 137.75 E | 400 m | Schweingruber, F.H. (2002-05-29): NOAA/WDS Paleoclimatology - Schweingruber - Khandiga River - PCOB - ITRDB RUSS057. NOAA National Centers for Environmental Information. https://doi.org/10.25921/bhed-3t82. Accessed 2023-11-16. |
| RUSS060 | PCOB | 64.92 N, 42.50 E | 70 m | Schweingruber, F.H. (2002-05-29): NOAA/WDS Paleoclimatology - Schweingruber - Leshukonskoe - PCOB - ITRDB RUSS060. NOAA National Centers for Environmental Information. https://doi.org/10.25921/whtf-an45. Accessed 2023-11-16. |
| RUSS071 | PCOB | 66.67 N, 82.33 E | 15 m | Schweingruber, F.H. (2002-05-28): NOAA/WDS Paleoclimatology - Schweingruber - Sidorovsk - PCOB - ITRDB RUSS071. NOAA National Centers for Environmental Information. https://doi.org/10.25921/2nps-by89. Accessed 2023-11-16. |
| RUSS072 | PCAB | 60.70 N, 51.38 E | 160 m | Schweingruber, F.H. (2002-05-29): NOAA/WDS Paleoclimatology - Schweingruber - Nyuchpas - PCAB - ITRDB RUSS072. NOAA National Centers for Environmental Information. https://doi.org/10.25921/g4qn-hj59. Accessed 2023-11-16. |
| RUSS085 | PCOB | 61.15 N, 136.57 E | 600 m | Schweingruber, F.H. (2002-05-28): NOAA/WDS Paleoclimatology - Schweingruber - Tschuchonoi River - PCOB - ITRDB RUSS085. NOAA National Centers for Environmental Information. https://doi.org/10.25921/ycr9-t605. Accessed 2023-11-16. |
| RUSS095 | PCOB | 62.60 N, 58.80 E | 340 m | Schweingruber, F.H. (2002-05-29): NOAA/WDS Paleoclimatology - Schweingruber - Ukyu - PCOB - ITRDB RUSS095. NOAA National Centers for Environmental Information. https://doi.org/10.25921/520e-t081. Accessed 2023-11-16. |
| RUSS096 | PCAB | 68.77 N, 32.80 E | 140 m | Schweingruber, F.H. (2002-05-28): NOAA/WDS Paleoclimatology - Schweingruber - Murmashi - PCAB - ITRDB RUSS096. NOAA National Centers for Environmental Information. https://doi.org/10.25921/1ca9-6z47. Accessed 2023-11-16. |
| RUSS097 | PCOB | 65.35 N, 69.52 E | 25 m | Schweingruber, F.H. (2002-05-28): NOAA/WDS Paleoclimatology - Schweingruber - Polui River Head B - PCOB - ITRDB RUSS097. NOAA National Centers for Environmental Information. https://doi.org/10.25921/3j6a-ms90. Accessed 2023-11-16. |
| RUSS104 | PCOB | 60.38 N, 57.12 E | 140 m | Schweingruber, F.H. (2002-05-28): NOAA/WDS Paleoclimatology - Schweingruber - Krasnovishersk - PCOB - ITRDB RUSS104. NOAA National Centers for Environmental Information. https://doi.org/10.25921/939r-0s16. Accessed 2023-11-16. |
| RUSS109 | PCOB | 63.65 N, 133.78 E | 200 m | Schweingruber, F.H. (2002-05-29): NOAA/WDS Paleoclimatology - Schweingruber - Baraii River - PCOB - ITRDB RUSS109. NOAA National Centers for Environmental Information. https://doi.org/10.25921/mcbd-0w55. Accessed 2023-11-16. |
| RUSS120 | PCOB | 61.27 N, 59.33 E | 670 m | Schweingruber, F.H. (2002-05-29): NOAA/WDS Paleoclimatology - Schweingruber - Molebny Kamen Ridge - PCOB - ITRDB RUSS120. NOAA National Centers for Environmental Information. https://doi.org/10.25921/qjxv-0402. Accessed 2023-11-16. |
| RUSS139 | PCOB | 50.15 N, 85.37 E | 1700 m | Schweingruber, F.H. (2002-05-28): NOAA/WDS Paleoclimatology - Schweingruber - Ust Koksa Valley (Altai) - PCOB - ITRDB RUSS139. NOAA National Centers for Environmental Information. https://doi.org/10.25921/0x6r-at18. Accessed 2023-11-16. |
| RUSS153 | PCOB | 59.78 N, 154.00 E | 80 m | Schweingruber, F.H. (2002-05-29): NOAA/WDS Paleoclimatology - Schweingruber - Jama river - PCOB - ITRDB RUSS153. NOAA National Centers for Environmental Information. https://doi.org/10.25921/v7az-gn67. Accessed 2023-11-16. |
| RUSS160 | PCOB | 70.27 N, 103.25 E | 130 m | Schweingruber, F.H. (2002-05-29): NOAA/WDS Paleoclimatology - Schweingruber - Kotuy River B - PCOB - ITRDB RUSS160. NOAA National Centers for Environmental Information. https://doi.org/10.25921/9rqc-b387. Accessed 2023-11-16. |
| RUSS161 | PCOB | 69.78 N, 119.12 E | 130 m | Schweingruber, F.H. (2002-05-28): NOAA/WDS Paleoclimatology - Schweingruber - Olenok River - PCOB - ITRDB RUSS161. NOAA National Centers for Environmental Information. https://doi.org/10.25921/1126-h015. Accessed 2023-11-16. |
| RUSS169 | PCOB | 56.50 N, 117.25 E | 1000 m | Schweingruber, F.H. (2002-05-29): NOAA/WDS Paleoclimatology - Schweingruber - Kodarpass (Baikal) - PCOB - ITRDB RUSS169. NOAA National Centers for Environmental Information. https://doi.org/10.25921/r2p9-s543. Accessed 2023-11-16. |
| RUSS174 | PCOB | 61.85 N, 93.40 E | 120 m | Schweingruber, F.H. (2002-05-28): NOAA/WDS Paleoclimatology - Schweingruber - White River, P.Tung. - PCOB - ITRDB RUSS174. NOAA National Centers for Environmental Information. https://doi.org/10.25921/wyvt-0r04. Accessed 2023-11-16. |
| RUSS175 | PCOB | 61.93 N, 95.15 E | 160 m | Schweingruber, F.H. (2002-05-28): NOAA/WDS Paleoclimatology - Schweingruber - Nirukda, P.Tung. - PCOB - ITRDB RUSS175. NOAA National Centers for Environmental Information. https://doi.org/10.25921/1ynb-pm42. Accessed 2023-11-16. |
| RUSS177 | PCOB | 63.13 N, 75.33 E | 150 m | Schweingruber, F.H. (2002-05-28): NOAA/WDS Paleoclimatology - Schweingruber - Nojabrsk west - PCOB - ITRDB RUSS177. NOAA National Centers for Environmental Information. https://doi.org/10.25921/8zev-j353. Accessed 2023-11-16. |
| SWIT169 | PCAB | 46.40 N, 7.43 E | 1900 m | Schweingruber, F.H. (2002-05-28): NOAA/WDS Paleoclimatology - Schweingruber - Simmental, Iffigenalp - PCAB - ITRDB SWIT169. NOAA National Centers for Environmental Information. https://doi.org/10.25921/2709-r005. Accessed 2023-11-16. |
| SWIT171 | PCAB | 46.43 N, 8.87 E | 1520 m | Schweingruber, F.H. (2002-05-28): NOAA/WDS Paleoclimatology - Schweingruber - Suaiza, TI - PCAB - ITRDB SWIT171. NOAA National Centers for Environmental Information. https://doi.org/10.25921/0k28-jd02. Accessed 2023-11-16. |
| SWIT173 | PCAB | 46.73 N, 9.08 E | 1520 m | Schweingruber, F.H. (2002-05-29): NOAA/WDS Paleoclimatology - Schweingruber - Obersaxen, Meierhof, GR - PCAB - ITRDB SWIT173. NOAA National Centers for Environmental Information. https://doi.org/10.25921/7w8m-3x42. Accessed 2023-11-16. |
| SWIT174 | PCAB | 46.60 N, 8.03 E | 1700 m | Schweingruber, F.H. (2002-05-29): NOAA/WDS Paleoclimatology - Schweingruber - Grindelwald Nord (N3) - PCAB - ITRDB SWIT174. NOAA National Centers for Environmental Information. https://doi.org/10.25921/msm3-yq32. Accessed 2023-11-16. |
| SWIT175* | PCAB | 46.65 N, 8.02 E | 1960 m | Schweingruber, F.H. (2002-05-29): NOAA/WDS Paleoclimatology - Schweingruber - Grindelwald Süd (S3) - PCAB - ITRDB SWIT175. NOAA National Centers for Environmental Information. https://doi.org/10.25921/4axb-8316. Accessed 2023-11-16. |
| SWIT176 | PCAB | 46.43 N, 7.82 E | 1900 m | Schweingruber, F.H. (2002-05-28): NOAA/WDS Paleoclimatology - Schweingruber - Lötschental oWG-SCH, CH - PCAB - ITRDB SWIT176. NOAA National Centers for Environmental Information. https://doi.org/10.25921/0pn2-vb21. Accessed 2023-11-16. |

**
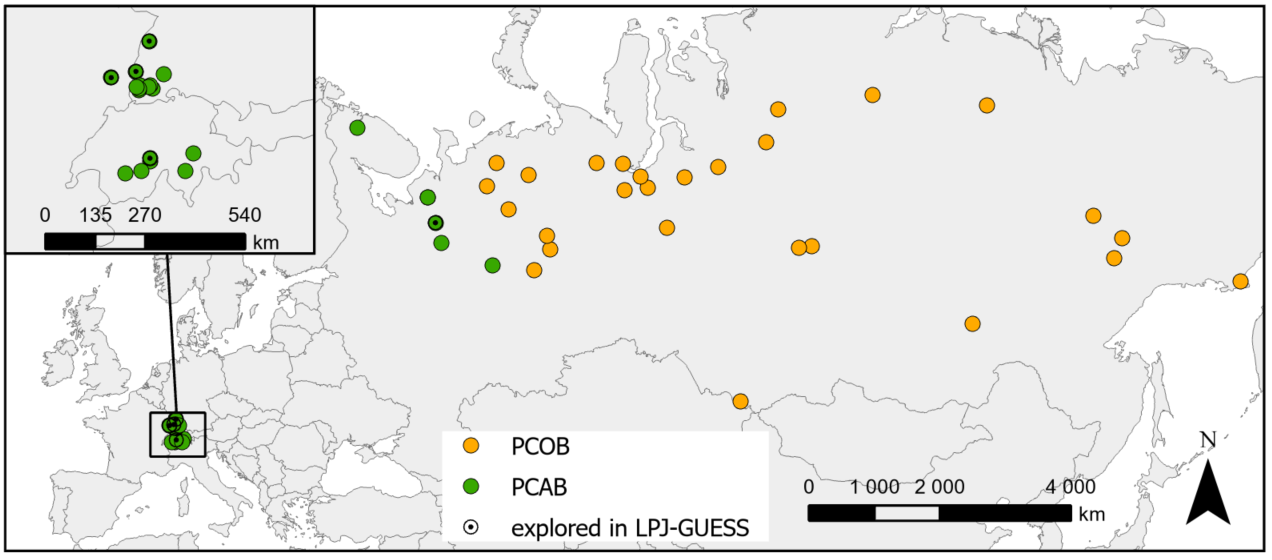
**

*Figure S1: Map of all locations included for the construction of the temperature-response function separated by the sub-species Norway Spruce (Picea abies, PCAB) and Siberian Spruce (Picea obovata, PCOB). The locations that were used to explore the effects in LPJ-GUESS are highlighted.*

- 1. Data processing

The data was first averaged per site and year for each of the variables and filtered to include only years within 1901-1999 resulting in overall 4725 site-year pairings. All site-year pairings were removed where at least one variable had a z-score greater than three.

Z-scores were calculated as:

$z = \frac{v_{s, y}- \underline{v}}{\sigma_{V}}$ *(Eq 1)*

where $v_{s, y}$is the value of a specific site and year to be z-scored, $\underline{v}$ is the mean and $\sigma_{V}$ is the standard deviation of that variable across all sites and years. Overall, 47 values were removed due to z-scoring leaving 4678 site-year pairings used for function building.

- 1. Monthly temperature-LWD analysis


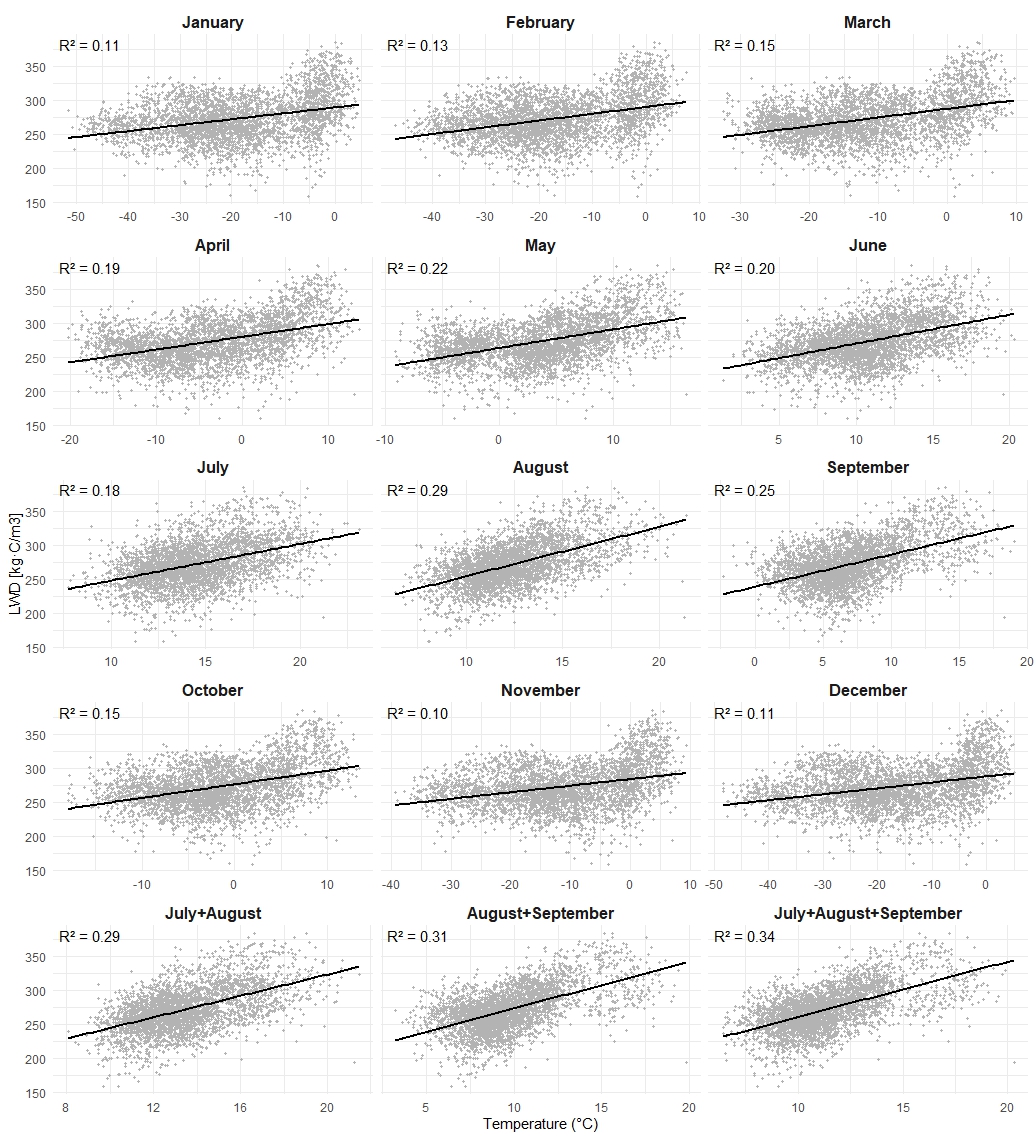


*Figure S2: All temperature variables tested for correlation with latewood density (LWD). All presented relationships were significant (p < 0.001), with the mean July–September temperature having the highest coefficient of determination (R² = 0.34). The coefficient of determination (R²) and p-values were obtained using the summary() function applied to linear models (lm()) in R (R Core Team, 2024).*


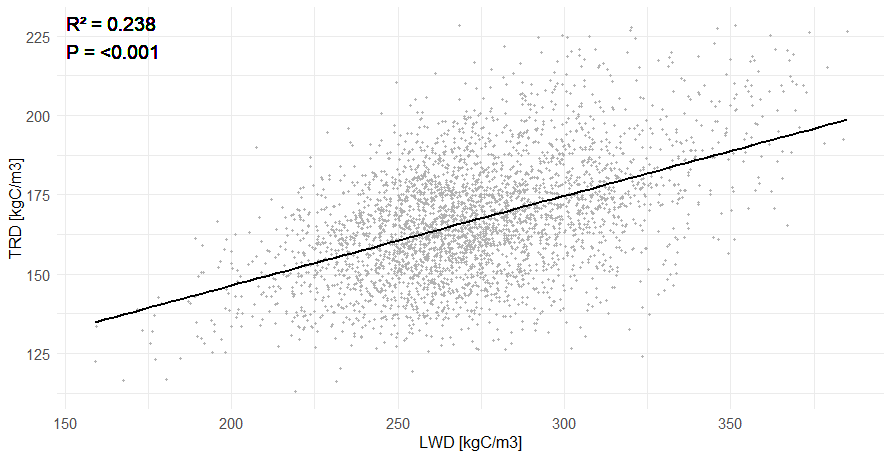


*Figure S3: Visualization of the relationship between latewood density (LWD) and tree ring density (TRD) from observed data used in this study (Table S1).*

*
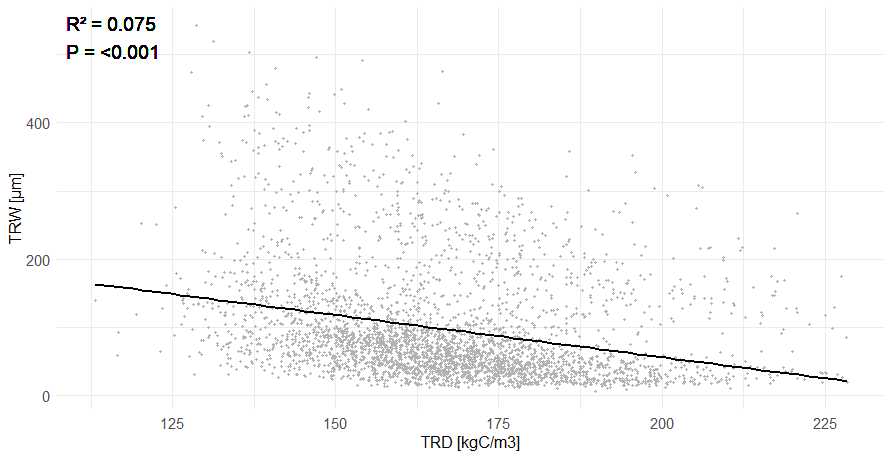
*

*Figure S4: Visualization of the relationship between tree ring density (TRD) and tree ring width (TRW) from observed data used in this study (Table S1).*


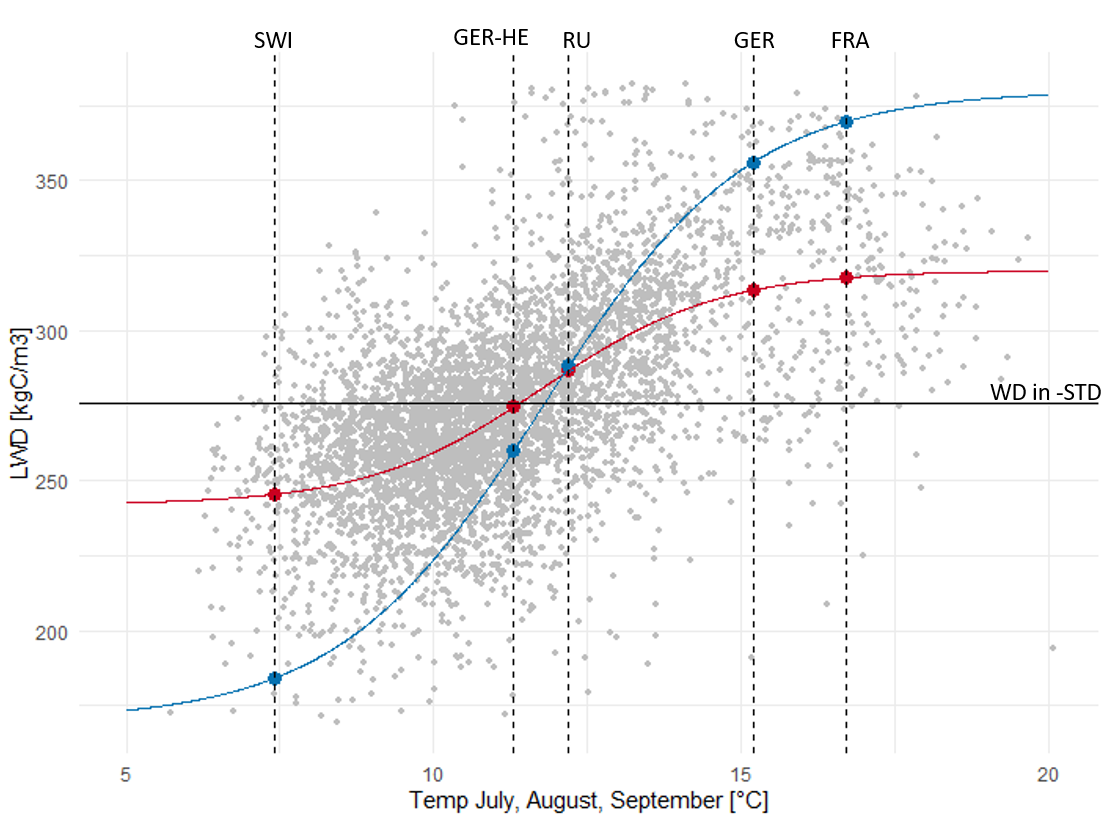


*Figure S5: Observations of the mean July+August+September temperature and latewood density (LWD) across the temperature-LWD continuum, used for constructing the response-function. Temperature observations stem from elevation adjusted CRU JRA data (University of East Anglia, 2020) and latewood density data stem from the International Tree Ring Databank (National Oceanic and Atmospheric Administration, 2022). Red curve: fLWD-Best; blue curve: fLWD-Range. Vertical dotted lines indicate the mean July+August+September temperature used in LPJ-GUESS for the five simulation sites. The horizontal line labelled WD in -STD indicates a latewood density that, in combination with the earlywood density parameter and the default earlywood-to-latewood ratio, corresponds to the fixed wood density parameter of 186 kgC/m³ in LPJ-GUESS-STD.*

***1.3.1 Comparison with fully empirical TRW-temperature response function***


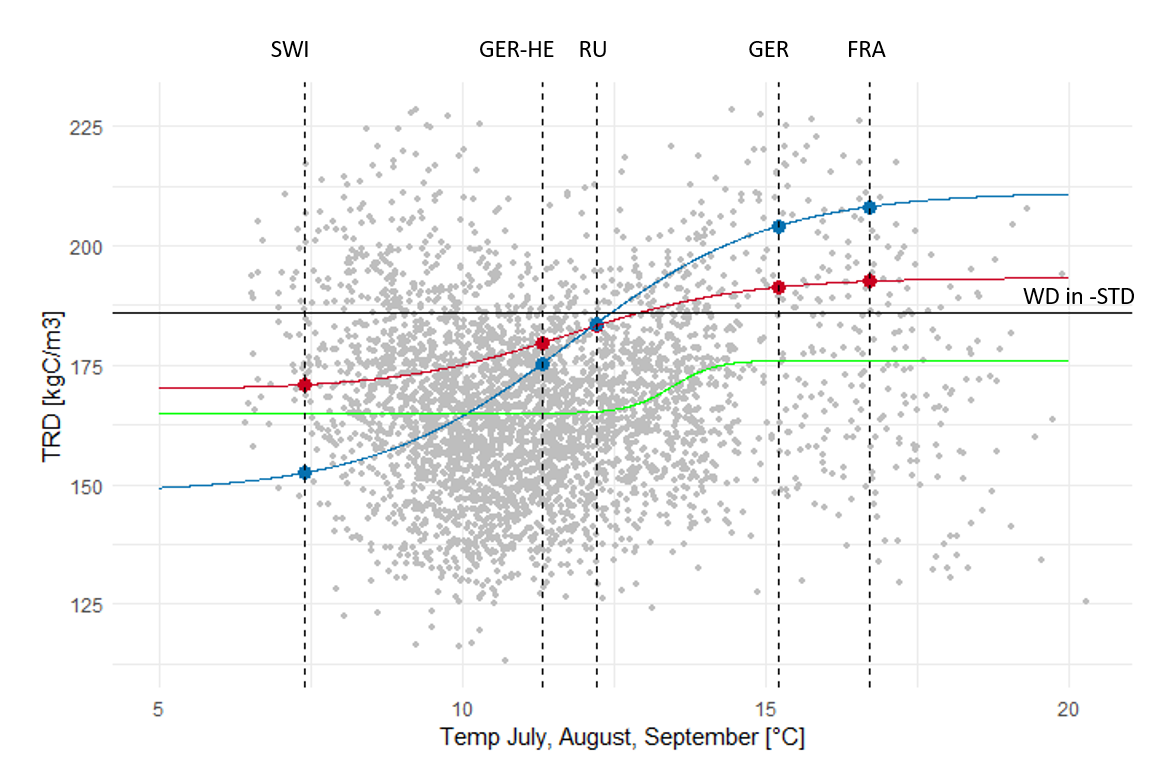


*Figure S6: Observations of the mean July+August+September temperature and tree ring density (TRD) across the temperature-LWD continuum, used for constructing the response-function. Temperature observations stem from elevation adjusted CRU JRA data (University of East Anglia, 2020) and tree ring density data stem from the International Tree Ring Databank (National Oceanic and Atmospheric Administration, 2022) TRD is a calculated value from measured earlywood density and width and latewood density and width by multiplying the two densities with the respective fraction of the ring width being earlywood or latewood. Tree ring density is only available for 39 of the observational sites as it is limited by the width data availability. Red curve: fLWD-Best; blue curve: fLWD-Range; green curve: fTRD.fTRD is a logistic growth curve fitted to the TRD observations following the same method as applied for fLWD-Best. Vertical dotted lines indicate the mean July+August+September temperature used in LPJ-GUESS for the five simulation sites. The horizontal line labelled WD in -STD indicates the fixed wood density parameter of 186 kgC/m³ in LPJ-GUESS-STD.*

**1.3.1 Why we did not use an empirically fitted TRD-temperature response function**

Direct TRD-temperature fitting (green line, Figure S6) would produce better agreement with current tree ring datasets and might appear more parsimonious by eliminating the need for earlywood density assumptions or the 70:30 EW:LW ratio. However, such empirical relationships conflate multiple environmental factors. Observed TRD-temperature correlations reflect not only temperature effects on wood formation but also covariation and compensation between temperature and water availability, growing season length, nutrient cycling, and competition, which all independently affect ring density.

Importantly, there is limited physiological basis for a direct causal link between temperature and TRD. As Björklund et al. (2017) demonstrated, temperature-density correlations often arise indirectly through covariance with other climatic drivers rather than through mechanistic temperature control on cell-wall thickening. When extrapolating to regions or time periods where these correlations differ—precisely what DGVMs must do under climate change scenarios—such confounded relationships become unreliable.

Our mechanistic approach isolates the temperature-LWD pathway, which has clear physiological basis (Cuny & Rathgeber, 2016), even though current simplifications (constant EW:LW ratio, fixed EW density) limit quantitative accuracy. This design choice prioritizes building a framework for progressive development: we can add water effects on EW density, phenological controls on EW:LW allocation, and nutrient influences as independent, testable mechanisms. In contrast, an empirical TRD function offers no clear path for mechanistic refinement.

For this proof-of-concept study exploring how wood density dynamics cascade through forest structure, the mechanistic approach better serves our goal of understanding process interactions rather than fitting current observations. The model produces TRD within observed ranges and captures qualitative temperature responses, which is sufficient for exploring the ecological consequences that are our primary focus.

*Table S2: Summary of data of different density variables given as kgC/m3. *Tree ring density is a calculated value from measured earlywood density and width and latewood density and width by multiplying the two densities with the respective fraction of the ring width being earlywood or latewood. Tree ring density is only available for 39 of the observational sites as it is limited by the width data availability.*

| **Variable** | **Minimum** | **Mean** | **Maximum** |
| --- | --- | --- | --- |
| Earlywood density | 101.4 | 139.1 | 182.2 |
| Latewood density | 169.6 | 274.6 | 382.0 |
| Tree ring density* | 113.1 | 166.9 | 213.5 |

- 1. Consistency of the LWD-temperature relationship across hierarchical levels

To ensure that the LWD-temperature relationships were not confounded by hierarchical structure (e.g. species or site), we examined the relationship between LWD and summer temperature across all organisational levels: individual trees, species and sites.

First, we visualised all the data points at the individual level (tree-year pairs) across the entire dataset using a 2D density plot to account for overplotting and reveal the overall trend (Figure S7). This analysis revealed a positive correlation between mean late summer temperature (July–September) and LWD.

Secondly, we assessed the relationship separately for each species (Figure S8). The positive trend remained consistent within both species, demonstrating that neither one species nor the difference between the species alone drives the observed trend in the entire dataset.

Thirdly, we evaluated the relationship at both the individual tree and aggregated site levels simultaneously (Figure S9). For each site, we plotted the slopes of a fitted linear function (using the lm() function in R Studio (R Core Team, 2024) for each individual tree, alongside the slopes for all individual trees within the site. This approach allowed us to assess whether the positive trend observed at larger scales was also reflected within sites and even within individual trees. The majority of individual trees showed positive slopes, and the aggregated site-level slopes were predominantly positive and significant. Deviations from this positive relationship may, for example, result from other limiting factors (such as water availability) or from measurement errors.

Together, these analyses confirm that the observed relationship is not a statistical artefact of data aggregation. Rather, the positive temperature–latewood density (LWD) relationship holds across levels of biological and spatial organisation, justifying the use of mean summer temperature at the site level as a predictor of mean annual LWD in further modelling.


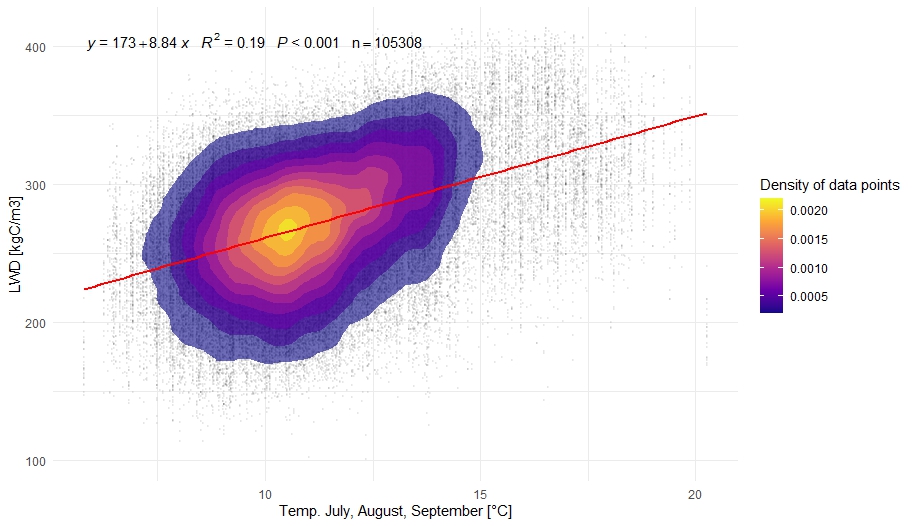


*Figure S7: Relationship between latewood density (LWD) and the mean temperature of July, August, and September across all individual trees and sites included in this study (see Table S1). A 2D density (using stat_density_2d() by R Core Team (2024)) overlay is included to account for overplotting and to illustrate regions of high data concentration.*


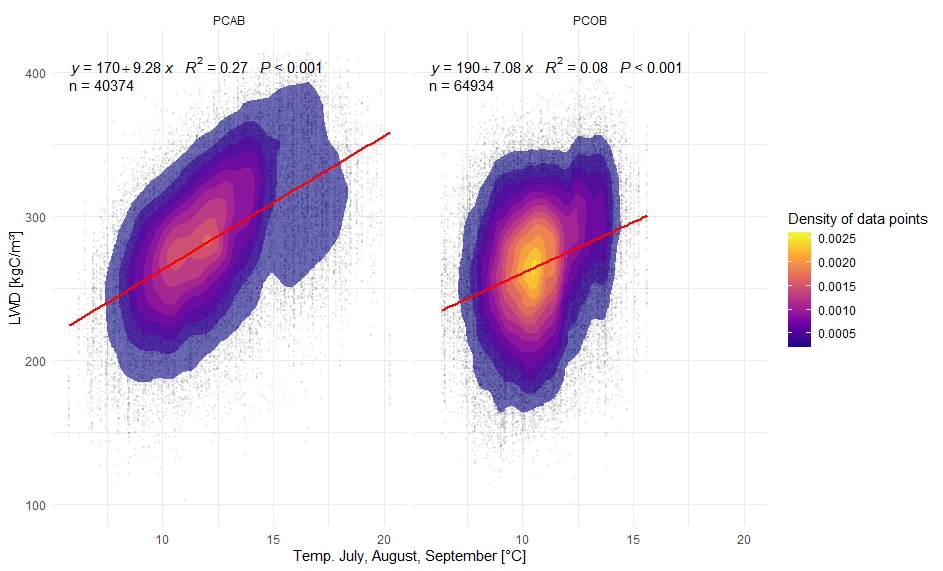


*Figure S8: Relationship between latewood density (LWD) and the mean temperature of July, August, and September across all individual trees and sites separate for the species Picea abies (PCAB) and Picea obovata (PCOB) included in this study (see Table S1). A 2D density overlay (using stat_density_2d() by R Core Team (2024)) is included to account for overplotting and to illustrate regions of high data concentration.*


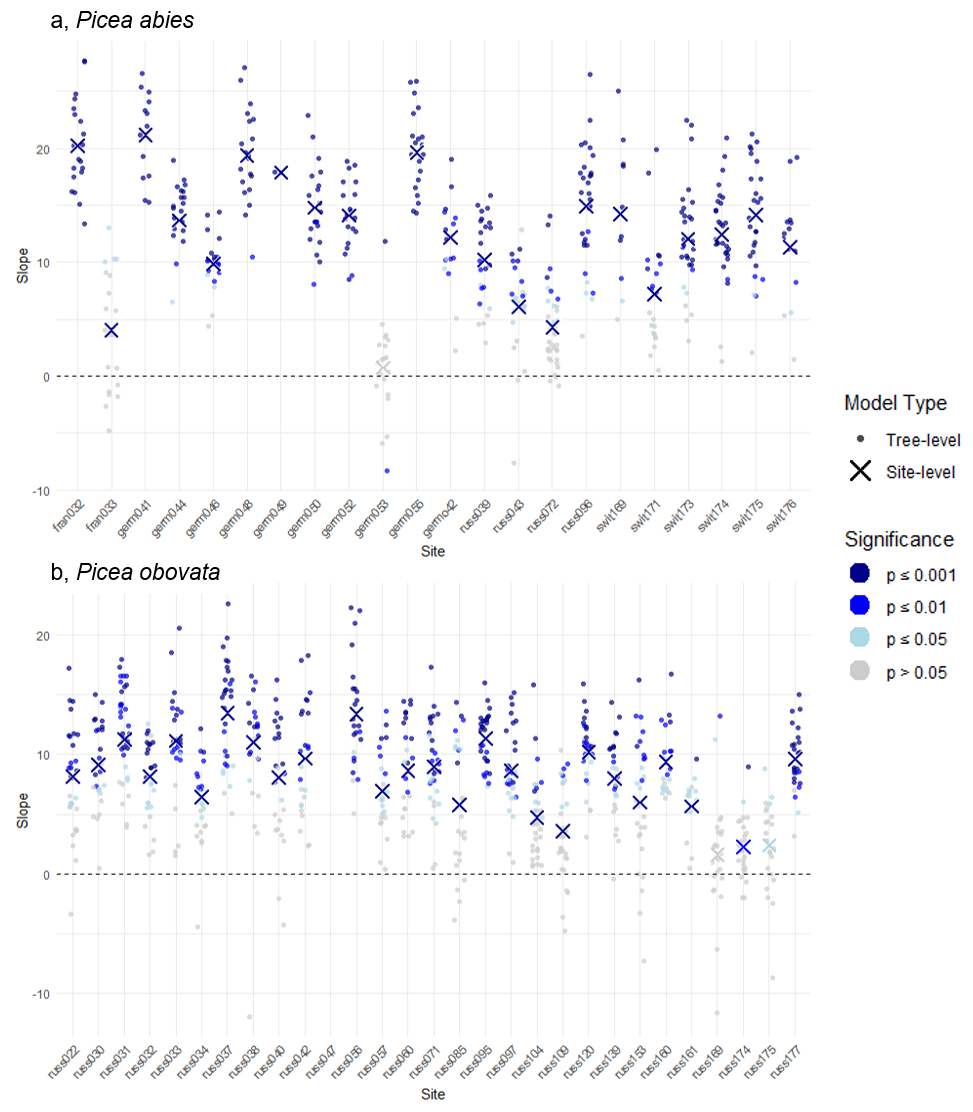


*Figure S9: Comparison of tree-level and site-level relationships between) mean summer temperature (July–September) and latewood density (LWD for each site, shown separately for a, Picea abies (PCAB) and b, Picea obovata (PCOB). Each point represents the slope of a linear model fitted to data of an individual tree, while crosses represent slopes from site-level regressions. Colours indicate significance levels (p-values), with darker shades representing more significant relationships. This visualization demonstrates that the majority of individual-level (70.29%) and site-level (94.12%) relationships are positive and statistically significant*

We proceeded as here demonstrated also to test the consistency of the relationships presented in Figure S7 and S8. A positive and significant relationship between latewood density and total ring density was found in 95.7% of individual trees and 97.8% of sites. A negative and significant relationship between tree ring density and tree ring width was found at 46.7% of individual trees and at 75.6% of the sites.

- 1. Changes to LPJ-GUESS-STD
     1. **Topographic effect on temperature**

In this study, we utilized the LPJ-GUESS model version with an enhanced topography module to simulate the elevation effect on temperature, which is a crucial influencing factor of wood density, vegetation structures, and dynamic (Björklund et al., 2017; Boakye et al., 2023; Cuny et al., 2019).

In this model version scheme, we used the relative elevation difference between the elevation of each sub-grid scale part and the gridcell mean elevation to obtain the temperature for each sub-grid scale part following the temperature-elevation decreasing rate (Dani et al., 2023; Xie et al., 2024; Yang et al., 2016).

$T_{sub-grid}= T_{gridcell}-\left( E_{sub-grid}- E_{gridcell} \right)*\frac{6.49}{1000}$

Where $T_{sub-grid}$ is the temperature of sub-grid scale part, $T_{gridcell}$ is the gridcell mean temperature read in from the CRU-JRA data inputs, $E_{sub-grid}$ is the elevation of sub-grid scale part, $E_{gridcell}$ is gridcell mean elevation.

- - 1. **Carbon allocation and integration of wood density in LPJ-GUESS**

To incorporate the dynamic wood density (WD) module into LPJ-GUESS, several changes were made to the STD model’s structure and allocation framework. The new formulation calculates height and height increment ($H_{n}$) as a function of mass (Eq 2), considering annual tree ring wood density (TRWD); and as a function of volume (Eq 3), taking into account the mean stem’s wood density (SWD). Tree height as a function of mass is given by:

$H_{n}= \frac{C_{s} + dBM - dC_{L}\cdot(1 + \frac{1}{L:R})}{C_{L} + dC_{L}}\cdot\frac{K_{LA:SA}}{{SWD}_{t} \cdot SLA}$ *(Eq 2)*

where *C_S_* and *C_L_* are the carbon bound in sapwood and in leaves respectively, $dBM$ is the change in biomass and *dC_L_* is the change of carbon in leaves. *L:R* is the ratio of carbon in leaves to carbon in roots. By that, $dBM - dC_{L}$ expresses the change in carbon in sapwood. $K_{LA:SA}$ is the leaf area to sapwood area parameter and $SLA$ is the specific leaf area. The STD version wood density parameter is replaced with ${SWD}_{t}$which is the mean stem wood density at a given year t.

The height as a function of volume is given as:

$H_{n}= \frac{C_{S} + C_{H}}{{SWD}_{t-1} \cdot\pi\cdot\frac{{DBH}^{4}}{2}} + \frac{dBM - dC_{L} \cdot(1 + \frac{1}{L:R})}{TRWD \cdot\pi\cdot\frac{{DBH}^{4}}{2} \cdot(\frac{4H}{K_{A2}\cdot K_{A3} \cdot{DBH}^{K_{A3}}}+1)}$ *(Eq 3)*

where $C_{H}$ is the carbon in heartwood and *DBH* the stem diameter at breast height. ${SWD}_{t-1}$ is the total wood density of the stem up to the previous year and replaces the wood density parameter in STD. $H$ is the height of the previous year, $TRWD$ is the wood density of the current year’s tree ring and $K_{A2}$ and $K_{A3}$ are PFT specific constants used in the allocation. The first addend expresses the mass of the tree of the prior year and the second addend expresses the mass gain of the current year. By balancing Eq. 2 and Eq. 3 the leaf carbon increment can be determined using an optimisation routine. Based on the leaf-to-root (*L:R*) parameter, the carbon root increment can be calculated. The sapwood increment is the carbon not utilised for leaf and root formation.

Dynamic wood density is updated annually for trees starting at the age of 5 and influences stem wood density, given by:

${SWD}_{t}= \frac{(C_{s} + C_{H}) \cdot{SWD}_{t -1} + dC_{s}\cdot TRWD}{C_{s} + C_{H} + dC_{S}}$ *(Eq 4)*

where *C_S_* and *C_H_* are the carbon in wood from the previous year and $dC_{s}$ the newly acquired carbon in sapwood. As both $dC_{s}$and ${SWD}_{t}$ require update and are mutually dependent on each other, the carbon allocation scheme in LPJ-GUESS-WD is executed twice. The first execution of the allocation happens after the calculation of TRWD but assumes the same total stem wood density of the previous and current year (${SWD}_{t-1}$ = ${SWD}_{t}$). With the resulting sapwood increment ($dC_{s}$) is the total trunk density updated (Eq. 3). The allocation is updated again with the updated stem wood density. If the results for sapwood increment from the two executions differ by more than 25%, the allocation is executed a third time.

- 1. Explored sites in LPJ-GUESS

*Table S3: Descriptive information of exploration sites in the LPJ-GUESS runs with mean temperature of the period July-September (mean Temp JAS) as a mean from the 30 year repeating climate and the mean wood density from the best-fitting (mean WD-Best) and range-covering function (mean WD-Range) resulting from the 30 year mean climate.*

| **Site** | **Location** | **Elevation** | **Mean**  **Temp JAS** | **Species** | **Mean**  **WD-Best** | **Mean**  **WD-Range** |
| --- | --- | --- | --- | --- | --- | --- |
| SWI | 46.65 N, 8.02 E | 1960 m | 7.44 °C | PCAB | 171 | 153 |
| GER-HE | 48.57 N, 8.00 E | 1080 m | 11.26 °C | PCAB | 180 | 175 |
| RU | 63.43 N, 43.55 E | 120 m | 12.21 °C | PCAB | 183 | 184 |
| GER | 48.08 N, 7.68 E | 440 m | 15.18 °C | PCAB | 191 | 204 |
| FRA | 47.98 N, 7.08 E | 230 m | 16.70 °C | PCAB | 193 | 208 |

1.6 Canopy closure stages

The canopy closure process in LPJ-GUESS can be approximated by a polynomial function, which stabilizes when the canopy is fully closed. For model setup 2, one ensemble member from the WD-Range was selected for each site based on the lowest RMSE relative to the ensemble mean. For the chosen ensemble member, we defined five distinct canopy closure stages at which temperature increases were applied.

We used a local polynomial regression with the loess() function in R (R Core Team, 2024) to model the relationship between age and crown area, from age zero up to the age of canopy closure plus 10 years. The age of canopy closure was defined as the point when crown area reaches at least 95% of the total possible area.

To determine when a temperature increase should be applied, we first identified the inflection point in the canopy closure process, which corresponds to the maximum of the first derivative of the polynomial function. The five canopy closure stages were then defined as follows:

- Establishment (0 years),
- Mid-Establishment to Inflection Point (half the age of the inflection point),
- Inflection Point (maximum of the first derivative of the polynomial function),
- Mid-Inflection to Canopy Closure Point (half the age between the inflection point and canopy closure),
- Canopy Closure (the age at which crown area reaches 95% of the total area).

Section 2: Supplementary results


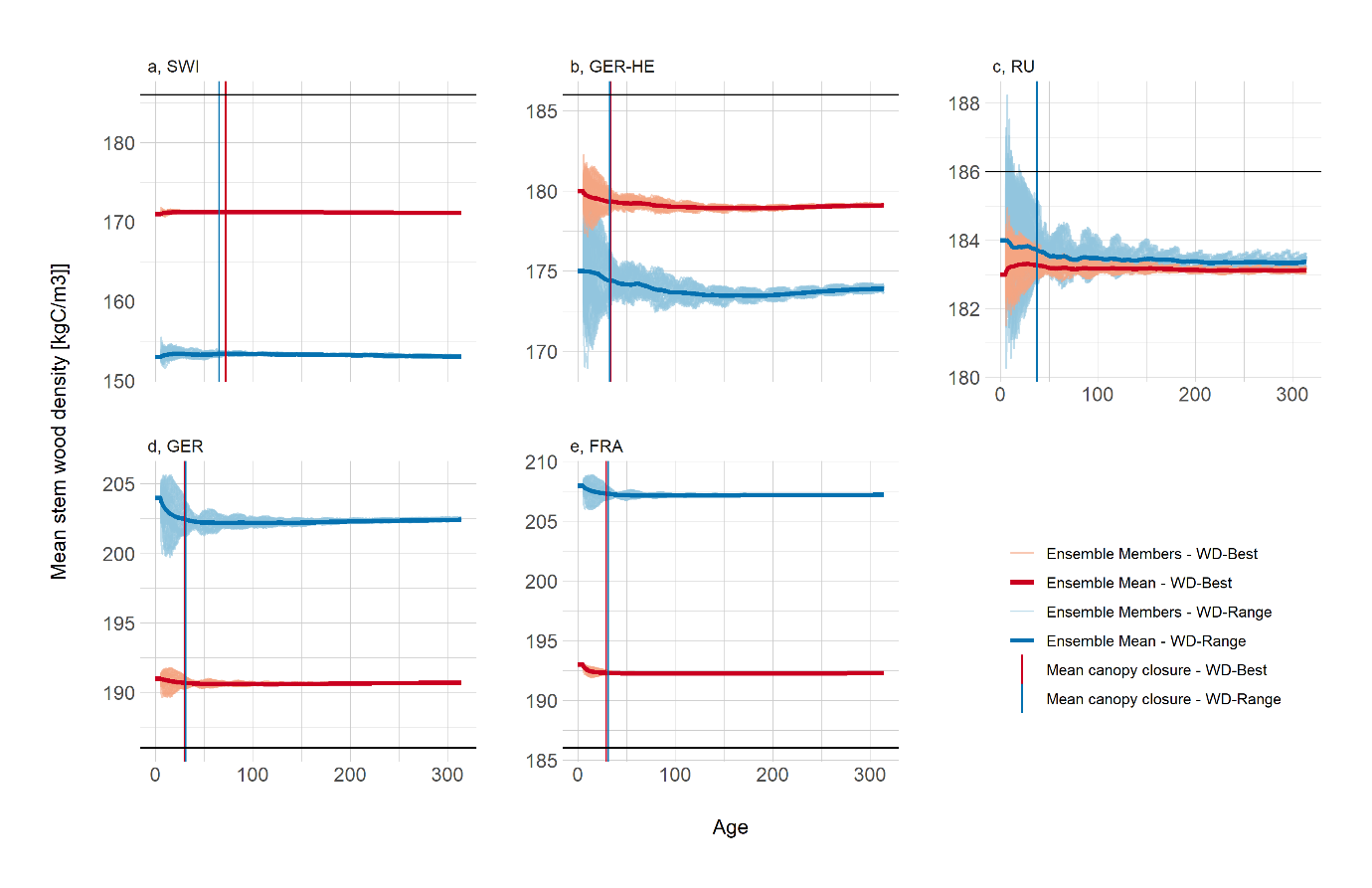


*Figure S10: Mean stem wood density for all five simulation sites and for both response-functions tested, as emergent from the climate and from productivity, emergent stem width increments, put in context with the constant parameter value for wood density in LPJ-GUESS-STD (black line). Shaded areas represent the range of values from climate ensemble members.*


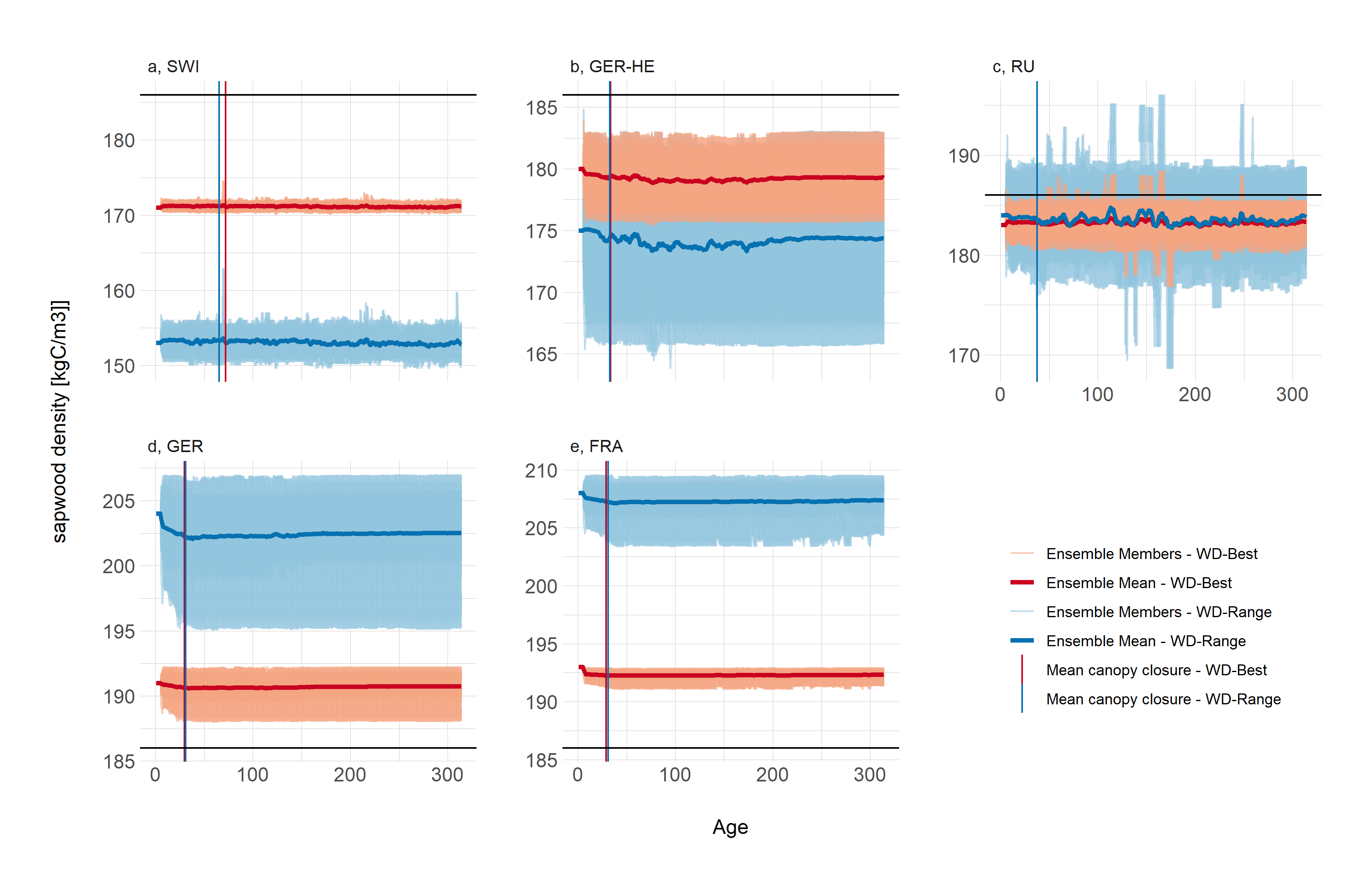


*Figure S11: Mean sapwood density for all five simulation sites and for both response-functions tested, as emergent from the climate and from productivity, emergent stem width increments, put in context with the constant parameter value for wood density in LPJ-GUESS-STD (black line). Shaded areas represent the range of values from climate ensemble members.*


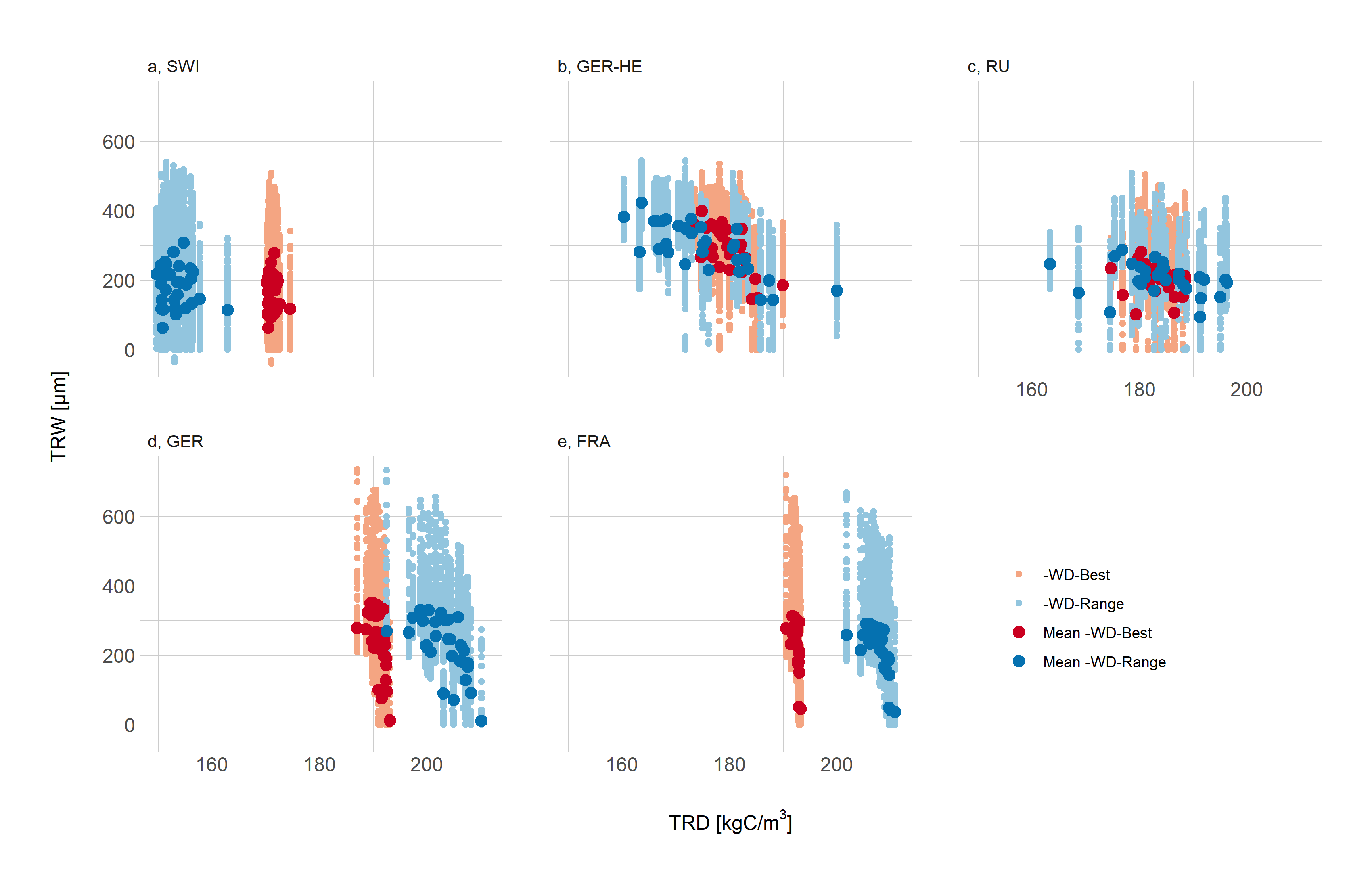


*Figure S12: Relationship between the mean annual tree ring density (TRD) and the annual tree ring width (TRW) for both the best fitting late wood density function (-WD-Best) and the wood density function covering the full range of observed latewood densities (-WD-Range).*


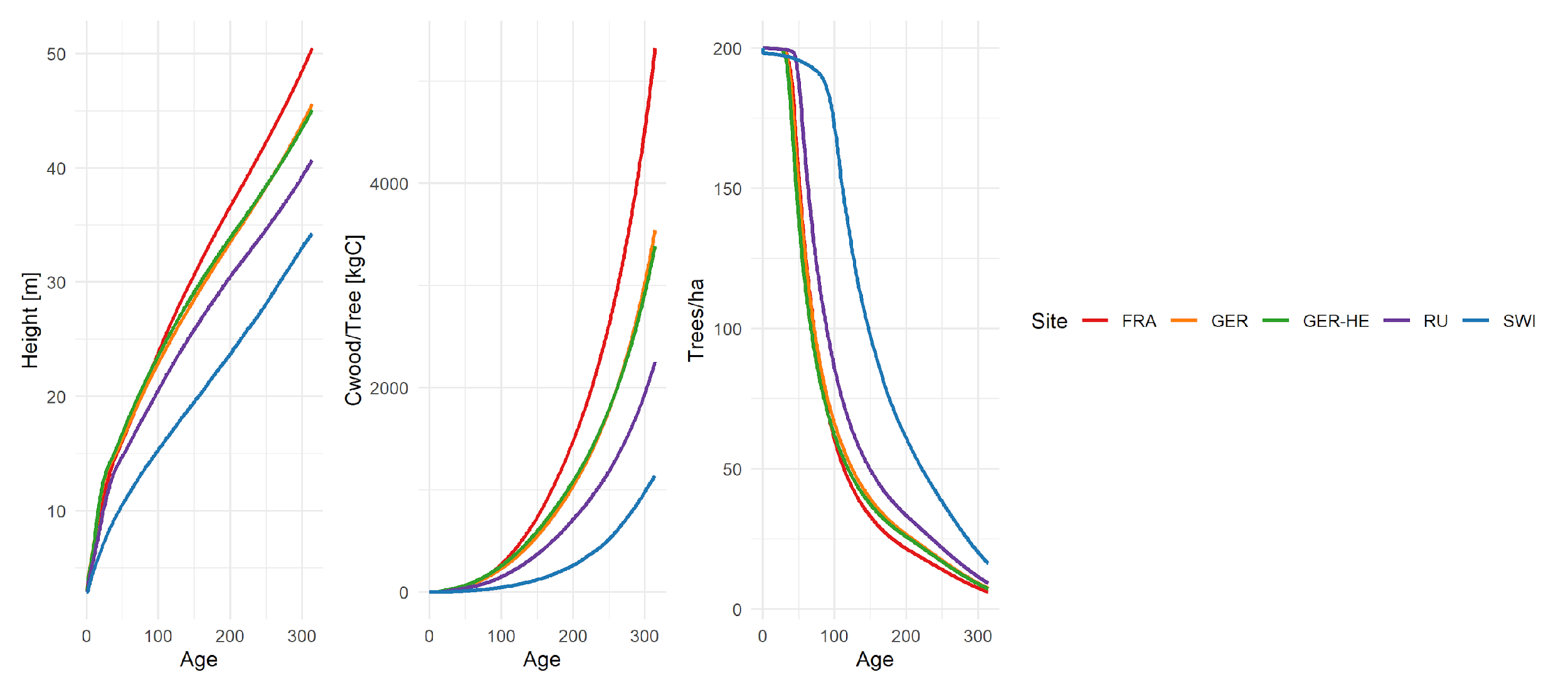


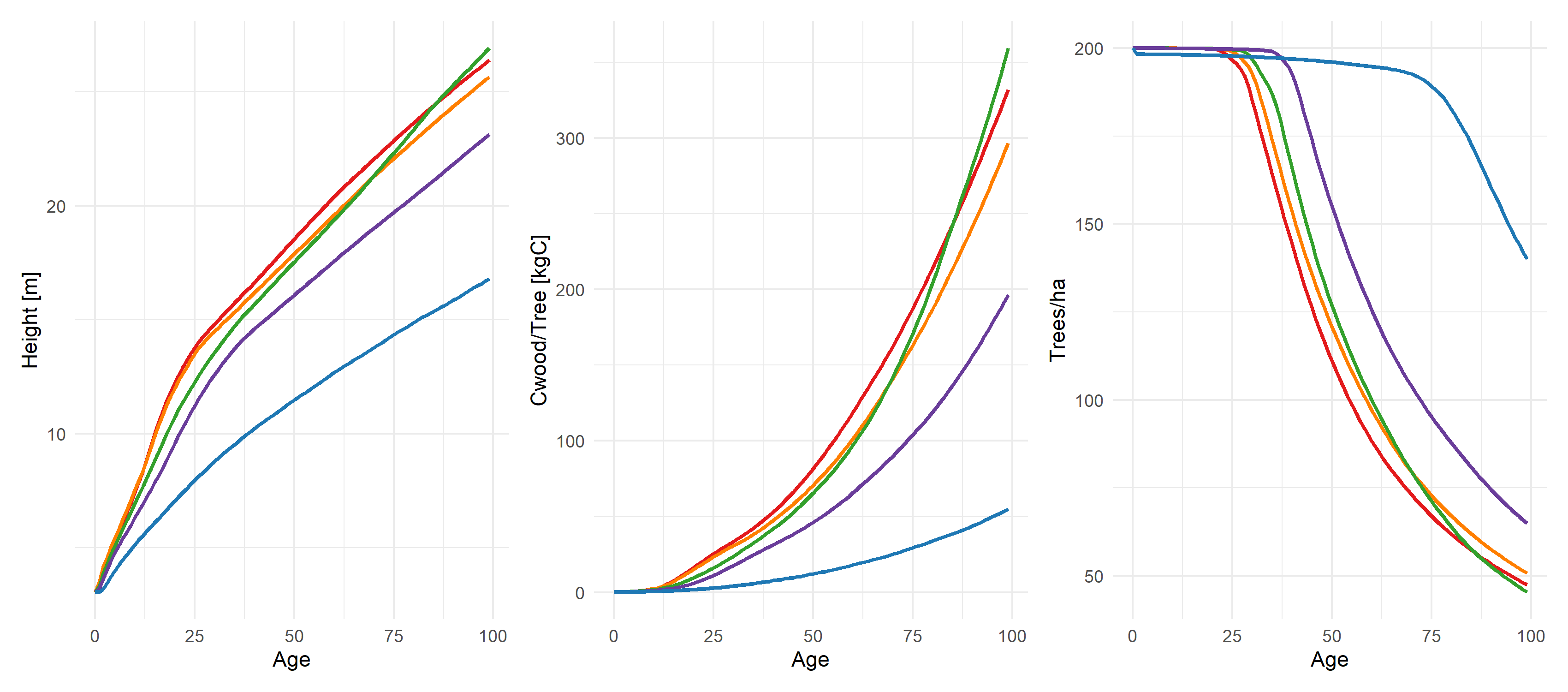


*Figure S13: Site-to-site variations in LPJ-GUESS-STD for mean height (Height), mean carbon mass in wood per individual tree (Cwood/Tree) and number of individuals (Trees/ha) over the time of regrowth (first 100 years).*

*Table S4: Absolute values of carbon in wood per tree (Cwood/Tree), Tree mean height (Height), Trees per hectare (Trees/ha) and Carbon in wood per m^2^ (Cwood/m^2^) at the age of 100 at the five simulation sites for the standard version of LPJ-GUESS (LPJ-GUESS-STD) and the two versions including dynamic wood density (LPJ-GUESS-WD) using the best-fit response function (LPJ-GUESS-WD-Best) and the range-covering response function (LPJ-GUESS-WD-Range). Blue highlights the site with the minimum and red the site with the maximum value across all sites respectively. Max Δ is the difference between these values.*

| **Cwood/Tree [kgC]** | **SWI** | **GER-HE** | **RU** | **GER** | **FRA** | **Max Δ** |
| --- | --- | --- | --- | --- | --- | --- |
| **LPJ-GUESS-STD** | 54.97 | 359.38 | 196.32 | 296.57 | 331.87 | 304.41 |
| **LPJ-GUESS-WD-Best** | 59.83 | 368.18 | 199.79 | 292.44 | 321.37 | 308.35 |
| **LPJ-GUESS-WD-Range** | 67.97 | 376.03 | 199.50 | 281.52 | 298.46 | 308.08 |

| **Height [m]** | **SWI** | **GER-HE** | **RU** | **GER** | **FRA** | **Max Δ** |
| --- | --- | --- | --- | --- | --- | --- |
| **LPJ-GUESS-STD** | 16.79 | 26.91 | 23.13 | 25.64 | 26.38 | 10.12 |
| **LPJ-GUESS-WD-Best** | 17.51 | 27.33 | 23.32 | 25.40 | 25.95 | 9.82 |
| **LPJ-GUESS-WD-Range** | 18.59 | 27.68 | 23.30 | 24.79 | 25.00 | 9.09 |

| **Trees/ha** | **SWI** | **GER-HE** | **RU** | **GER** | **FRA** | **Max Δ** |
| --- | --- | --- | --- | --- | --- | --- |
| **LPJ-GUESS-STD** | 140.03 | 45.33 | 64.92 | 50.75 | 47.45 | 94.70 |
| **LPJ-GUESS-WD-Best** | 126.67 | 43.68 | 63.67 | 51.95 | 49.36 | 82.88 |
| **LPJ-GUESS-WD-Range** | 109.79 | 42.36 | 63.79 | 55.05 | 53.95 | 67.43 |

| **Cwood/m^2^ [kgC/m^2^]** | **SWI** | **GER-HE** | **RU** | **GER** | **FRA** | **Max Δ** |
| --- | --- | --- | --- | --- | --- | --- |
| **LPJ-GUESS-STD** | 7.63 | 16.21 | 12.73 | 15.01 | 15.70 | 8.58 |
| **LPJ-GUESS-WD-Best** | 7.52 | 16.00 | 12.70 | 15.14 | 15.81 | 8.48 |
| **LPJ-GUESS-WD-Range** | 7.41 | 15.84 | 12.70 | 15.45 | 16.05 | 8.64 |


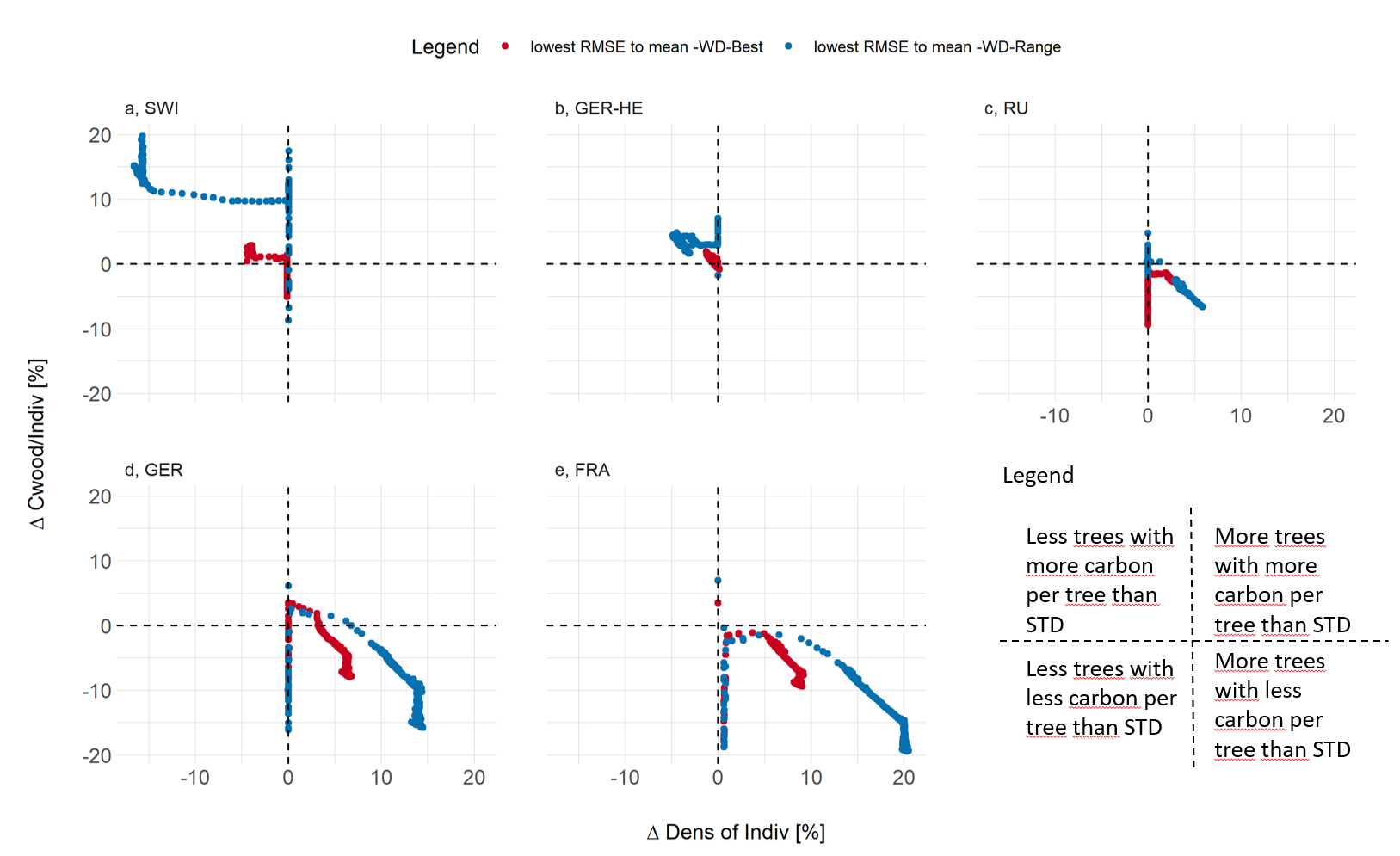


*Figure S14: The density of individuals (indiv/m2) related to the carbon stored in wood of individual trees as relative differences between LPJ-GUESS-STD and LPJ-GUESS-WD, for the best-fit (-WD-Best) and full-ranged (-WD-Range) temperature-response-curve; we focus on the ensemble members with the lowest RMSE to the ensemble mean of the two response functions.*


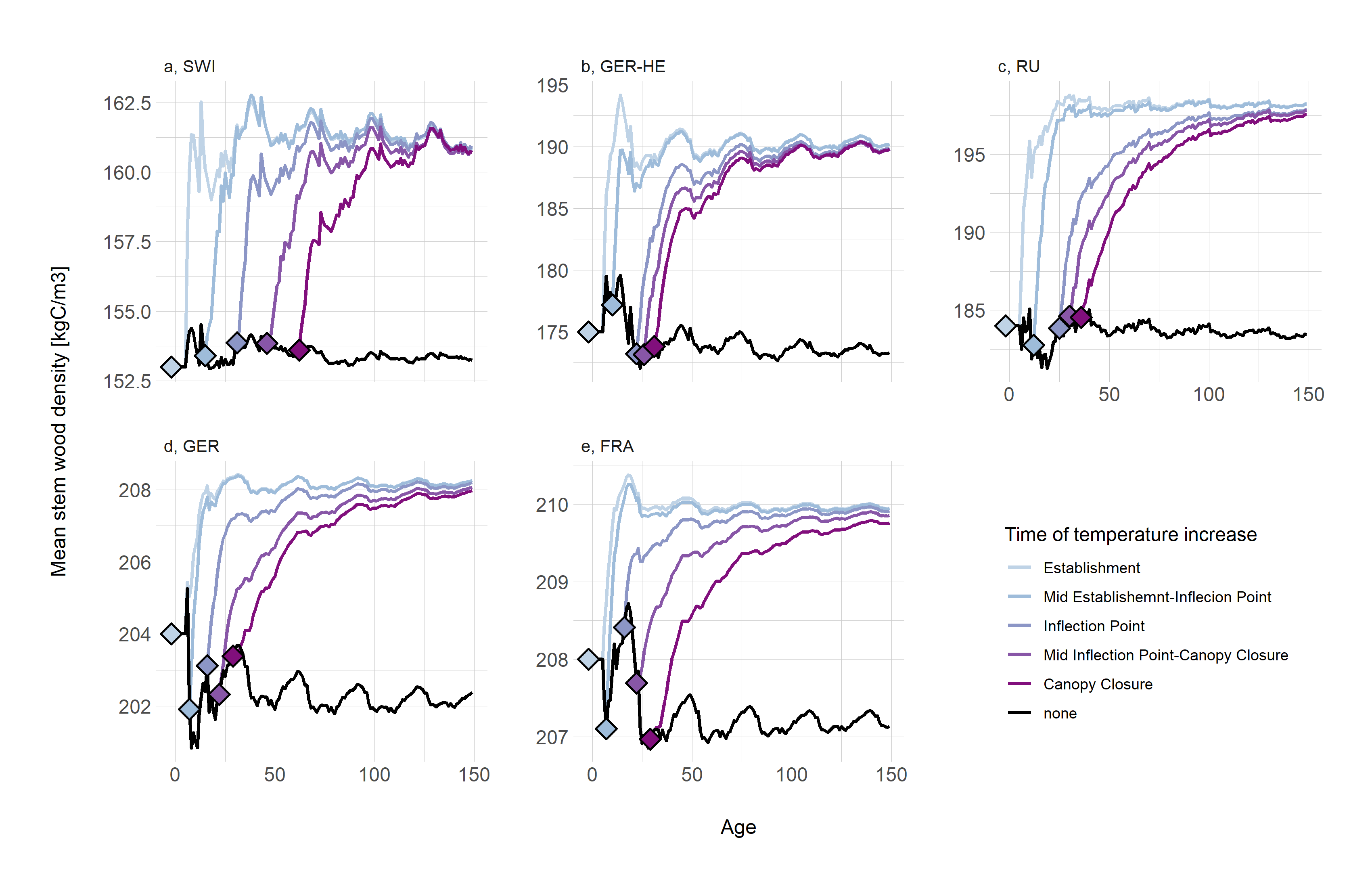


*Figure S15: Mean stem wood density for all five simulation sites, as emergent from the climate (and productivity), without temperature increase (black line) and temperature increase of 2 C at different stages of canopy closure*


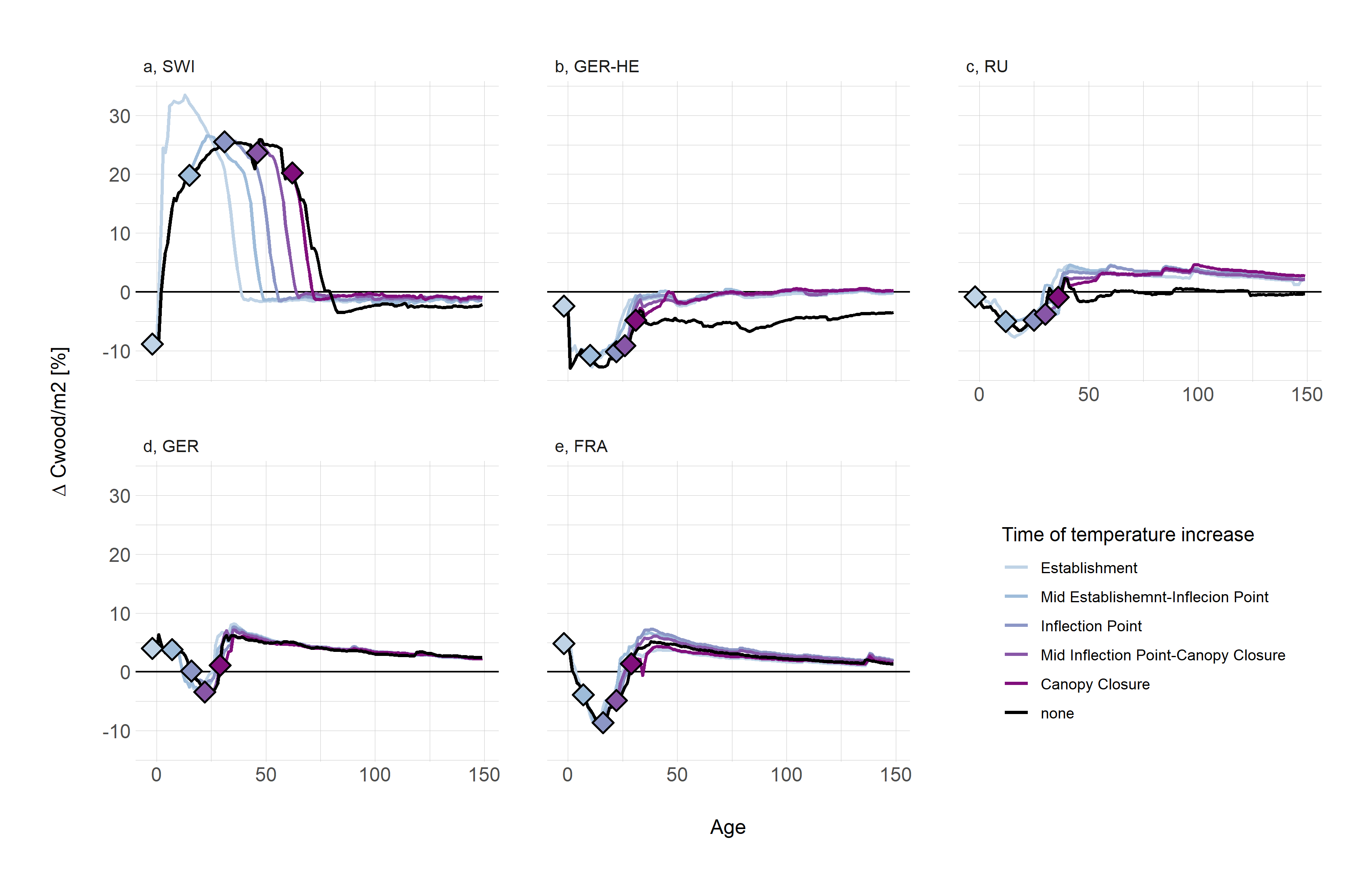


*Figure S16: Carbon in wood per m2 density for all five simulation sites, as emergent from the climate (and productivity), without temperature increase (black line) and temperature increase of 2 C at different stages of canopy closure*

References

Björklund, J., Seftigen, K., Schweingruber, F., Fonti, P., von Arx, G., Bryukhanova, M. V., Cuny, H. E., Carrer, M., Castagneri, D., & Frank, D. C. (2017). Cell size and wall dimensions drive distinct variability of earlywood and latewood density in Northern Hemisphere conifers. New Phytologist, 216(3), 728-740. <https://doi.org/10.1111/nph.14639>

Boakye, E. A., Mvolo, C. S., & Stewart, J. (2023). Systematic Review: Climate and Non-Climate Factors Influencing Wood Density in the Boreal Zone. BioResources, 18(4), 8757-8770. <https://ojs.bioresources.com/index.php/BRJ/article/view/22885>

Cuny, H. E., Fonti, P., Rathgeber, C. B. K., von Arx, G., Peters, R. L., & Frank, D. C. (2019). Couplings in cell differentiation kinetics mitigate air temperature influence on conifer wood anatomy. Plant, Cell & Environment, 42(4), 1222-1232. <https://doi.org/10.1111/pce.13464>

Dani, R. S., Divakar, P. K., & Baniya, C. B. (2023). Diversity and composition of plants species along elevational gradient: research trends. Biodiversity and Conservation, 32(8), 2961-2980. <https://doi.org/10.1007/s10531-023-02638-3>

R Core Team. (2024). _R: A Language and Environment for Statistical Computing_. In R

754 Foundation for Statistical Computing. https://www.R-project.org/

Xie, Y., Shen, Z., Wang, T., Malanson, G. P., Peñuelas, J., Wang, X., Chen, X., Liang, E., Liu, H., Yang, M., Ying, L., Zhao, F., & Piao, S. (2024). Uppermost global tree elevations are primarily limited by low temperature or insufficient moisture. Global Change Biology, 30(4), e17260. https://doi.org/10.1111/gcb.17260

Yang, Z., Edward, H., & and Callaghan, T. V. (2016). Modelling surface‐air‐temperature variation over complex terrain around abisko, swedish lapland: uncertainties of measurements and models at different scales. Geografiska Annaler: Series A, Physical Geography, 93(2), 89-112. <https://doi.org/10.1111/j.1468-0459.2011.00005.x>
